# Distribution of aquaporins and sodium transporters in the gastrointestinal tract of a desert hare, *Lepus yarkandensis*

**DOI:** 10.1038/s41598-019-53291-2

**Published:** 2019-11-12

**Authors:** Jianping Zhang, Shuwei Li, Fang Deng, Buheliqihan Baikeli, Weijiang Yu, Guoquan Liu

**Affiliations:** 1 College of Life Science, Tarim University Alar, Xinjiang Province, 843300 People’s Republic of China; 2 Key Laboratory of Biological Resources Protection and Utilization in Tarim Basin, Tarim University Alar, Xinjiang Province, 843300 People’s Republic of China; 3 Department of Biochemistry and Molecular Biology, School of Laboratory Medicine, and Anhui Province Key Laboratory of Translational Cancer Research Bengbu Medical College Bengbu, Anhui Province, 233030 People’s Republic of China; 4 Department of Basic Veterinary Medicine, and Key Lab of Swine Genetics and Breeding and Agricultural Animal Breeding and Reproduction, College of Animal Science and Veterinary Medicine Huazhong Agricultural University Wuhan, Hubei Province, 430070 People’s Republic of China

**Keywords:** Animal physiology, Proteins

## Abstract

*Lepus yarkandensis* is a desert hare of the Tarim Basin in western China, and it has strong adaptability to arid environments. Aquaporins (AQPs) are a family of water channel proteins that facilitate transmembrane water transport. Gastrointestinal tract AQPs are involved in fluid absorption in the small intestine and colon. This study aimed to determine the distribution of AQPs and sodium transporters in the gastrointestinal tract of *L. yarkandensis* and to compare the expression of these proteins with that in *Oryctolagus cuniculus*. Immunohistochemistry was performed to analyse the cellular distribution of these proteins, and the acquired images were analysed with IpWin32 software. Our results revealed that AQP1 was located in the colonic epithelium, central lacteal cells, fundic gland parietal cells, and capillary endothelial cells; AQP3 was located in the colonic epithelium, small intestinal villus epithelium, gastric pit and fundic gland; AQP4 was located in the fundic gland, small intestinal gland and colonic epithelium; and epithelial sodium channel (ENaC) and Na^+^-K^+^-ATPase were located in the epithelial cells, respectively. The higher expression levels of AQP1, AQP3, ENaC and Na^+^-K^+^-ATPase in the colon of *L. yarkandensis* compared to those in *O. cuniculus* suggested that *L. yarkandensis* has a higher capacity for faecal dehydration.

## Introduction

*Lepus yarkandensis* (Yarkand hare) inhabits the arid environments of the Tarim Basin, southern Xinjiang Uygur Autonomous Region of northwestern China, around edge of Takla Makan Desert^1^. The Tarim Basin is extremely dry, with annual precipitation levels below 100 mm—mostly below 50 mm—and water availability is very limited or scarce^2^. Due to its extended habitation in this arid environment, *L. yarkandensis* faces the sizeable challenge of maintaining salt and water homeostasis. *L. yarkandensis* is efficient at adaptability to the environment; for example, its size is smaller to reduce water loss, its coat colour is very close to that of its habitat, and its auditory organs are very well developed, with ears up to 10 cm longer than those of other rabbits. In addition, the Na^+^ levels are higher and Ca^2+^ levels are lower in the blood of *L. yarkandensis* than in the blood of *Oryctolagus cuniculus*, suggesting the strong adjustment ability of *L. yarkandensis* in maintaining body water. However, the molecular mechanism of *L. yarkandensis* water conservation is unclear.

Aquaporins (AQPs) are a family of water channel proteins that facilitate transmembrane water transport and play a significant role in the regulation of water homeostasis^3–5^. These proteins are present in various organs and tissues in mammals and are highly expressed in various tissues, such as the kidney, digestive tract, eye and heart, where rapid regulation of body fluid secretion and water absorption is necessary^4,6^. Besides the kidney, the digestive tract is the organ with the highest amounts of body fluid absorption and secretion; the amount of liquid transported in the human digestive tract is 8 to 10 L per day^7^. The water from food (approximately 2 L/day) and digestive juices (approximately 7 L/day) enters the digestive tract, and this fluid is almost entirely absorbed by the small intestine and colon. Water transport is physiologically crucial for the gastrointestinal tract in maintaining body water homeostasis and ensuring digestive and absorptive functions^8^. The importance of AQPs in the gastrointestinal tract is evident; several AQPs—AQP1, AQP3, AQP4 and AQP8-11—are found in the gastrointestinal tract of humans, rats and mice^8–26^. Mice with knockout of various AQPs have provided direct evidence that gastrointestinal tract AQPs are involved in the secretion of saliva, processing of dietary fat, and fluid transport in the small intestine and colon^7,27–30^. Despite the finding of several AQPs in the human, rat and mouse gastrointestinal tracts, very few studies have addressed the distribution of AQPs in rabbits and hares, especially those living in an arid desert environment.

Water transport through AQPs is driven by an osmotic gradient usually created by transcellular sodium transport. The general paradigm for water movement in the gastrointestinal tract is that active Na^+^ transport drives osmotic water transport. Na^+^ entry is conductive and mediated by apically located epithelial sodium channels (ENaCs), and Na^+^ exit is mediated through basolateral Na^+^-K^+^-ATPases^31^. Hummler and colleagues^32^ showed that mice deficient in ENaC died within 40 h after birth because of an inability for fluid clearance in the lung. Matalon and colleagues^33^ found that amiloride (inhibits ENaC) and ouabain (inhibits Na^+^-K^+^-ATPase) greatly reduced the rate of water clearance. Therefore, water absorption in the gastrointestinal tract is likely dependent upon both AQPs and sodium transporters.

We aimed to determine the distribution of AQPs and sodium transporters in different segments of the gastrointestinal tract of a desert hare, *L. yarkandensis*. *O. cuniculus* is a rabbit living in mesic environment and the neighbor-joining topology based on the 12S rDNA sequences showed that the relationship between *O. cuniculus* and *L. yarkandensis* is as high as 98%^34^. Thus, we compared the expression of these proteins with that in *O. cuniculus*. The comparative study of AQP expression/localization in the gastrointestinal tract between a xeric mammalian species and a mesic species is useful for understanding the physiological roles of AQPs under arid environmental conditions.

## Results

### Histology of the stomach, small intestine and large intestine of *L. yarkandensis*

**Figure 1:**
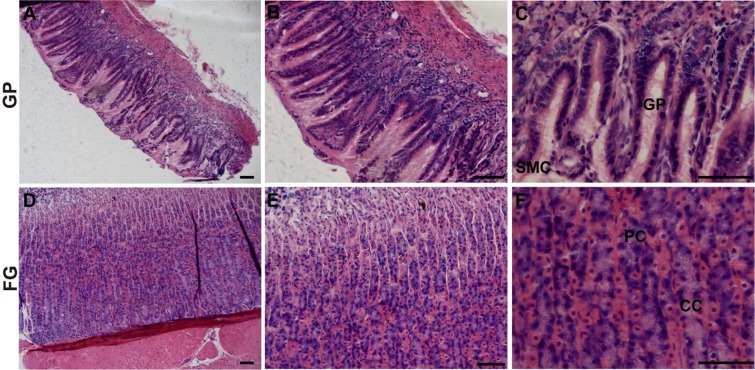
Histology of the *L. yarkandensis* stomach. Representative images of haematoxylin and eosin staining of the *L. yarkandensis* stomach (scale bar for **A** and **D**: 200 μm; scale bar for **B** and **E**: 100 μm; scale bar for **C** and **F**: 50 μm). Gastric pit (GP), surface mucous cell (SMC), fundic gland (FG), parietal cell (PC), chief cell (CC).

Since the histological structure of the *L. yarkandensis* gastrointestinal tract has not been reported, we used haematoxylin and eosin staining to observe this structure. After the stomach, small intestine and large intestine of *L. yarkandensis* were fixed with 4% paraformaldehyde, paraffin sections of these tissues were stained with haematoxylin and eosin. The gastric mucosal epithelium of *L. yarkandensis* is mainly composed of surface mucous cells (SMCs), and some parts of the epithelium are depressed to form many gastric pits (GPs) (Fig. 1A–C). The fundic gland of *L. yarkandensis* can be divided into the neck, body and bottom. The neck is connected to the gastric pits, the body is relatively long, and the bottom extends to the mucosal muscle. The fundic glands are mainly composed of parietal cells and chief cells (Fig. 1D–F). Parietal cells (PC) show a pink colour when stained with haematoxylin and eosin; they have a large volume and their nuclei are round and located at the middle of the cell. Chief cells (CC) are blue when stained with haematoxylin and eosin, and their nuclei are round and located at the base of the cell.

**Figure 2:**
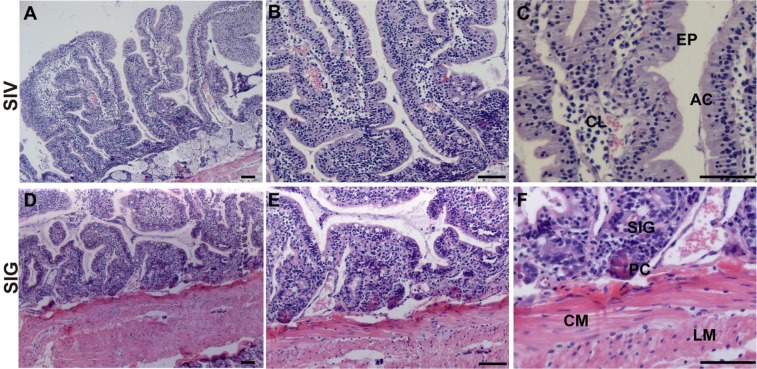
Histology of the *L. yarkandensis* small intestine. Representative images of haematoxylin and eosin staining of the *L. yarkandensis* small intestine (scale bar for **A** and **D**: 200 μm; scale bar for **B** and **E**: 100 μm; scale bar for **C** and **F**: 50 μm). Small intestinal villus (SIV), central lacteal (CL), epithelium (EP), absorptive cells (AC), small intestinal gland (SIG), Paneth cell (PC), circular muscle (CM), longitudinal muscle (LM).

The wall of the small intestine of *L. yarkandensis* is divided into the mucosa, submucosa, muscular layer and serosa, progressing from the inside to the outside. There are many plicas and intestinal villi on the small intestinal mucosa. The small intestinal mucosa can be divided into the epithelium (EP), lamina propria and muscularis mucosa. The epithelium is mainly composed of columnar absorptive cells (AC), and the lamina propria of the intestinal villi has a central lacteal (CL) (Fig. 2A–C). The epithelial root of the intestinal villi is subdivided into the lamina propria to form the small intestinal gland (SIG), and Paneth cells (PC) distributed at the bottom of the small intestinal gland are visible (Fig. 2D–F).

**Figure 3:**
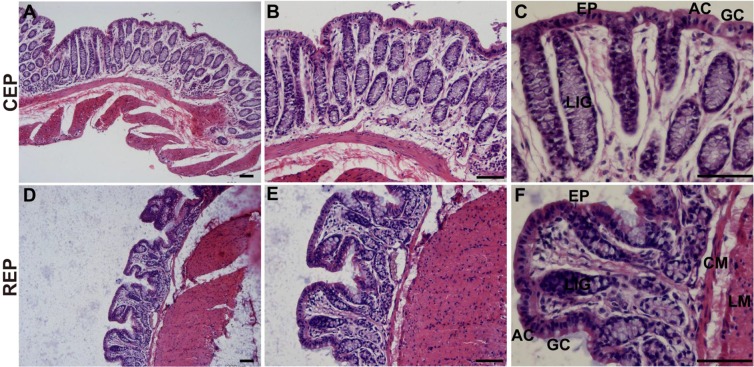
Histology of the *L. yarkandensis* large intestine. Representative images of haematoxylin and eosin staining of the *L. yarkandensis* large intestine (scale bar for **A** and **D**: 200 μm; scale bar for **B** and **E**: 100 μm; scale bar for **C** and **F**: 50 μm). Colonic epithelium (CEP), epithelium (EP), absorptive cells (AC), goblet cell (GC), large intestinal gland (LIG), rectal epithelium (REP), circular muscle (CM), longitudinal muscle (LM).

The wall of the large intestine of *L. yarkandensis* is divided into the mucosa, submucosa, muscular layer and serosa. There are no wrinkles and intestinal villi in the large intestinal mucosa; the mucosal epithelium (EP) has many absorptive cells (AC) and goblet cells (GC); the large intestinal gland (LIG) is developed, long and straight, without Paneth cells; and the muscular layer of the large intestine is developed (Fig. 3A–F).

### Localization of AQP1, AQP3 and AQP4 in the *O. cuniculus* and *L. yarkandensis* gastrointestinal tracts

**Figure 4:**
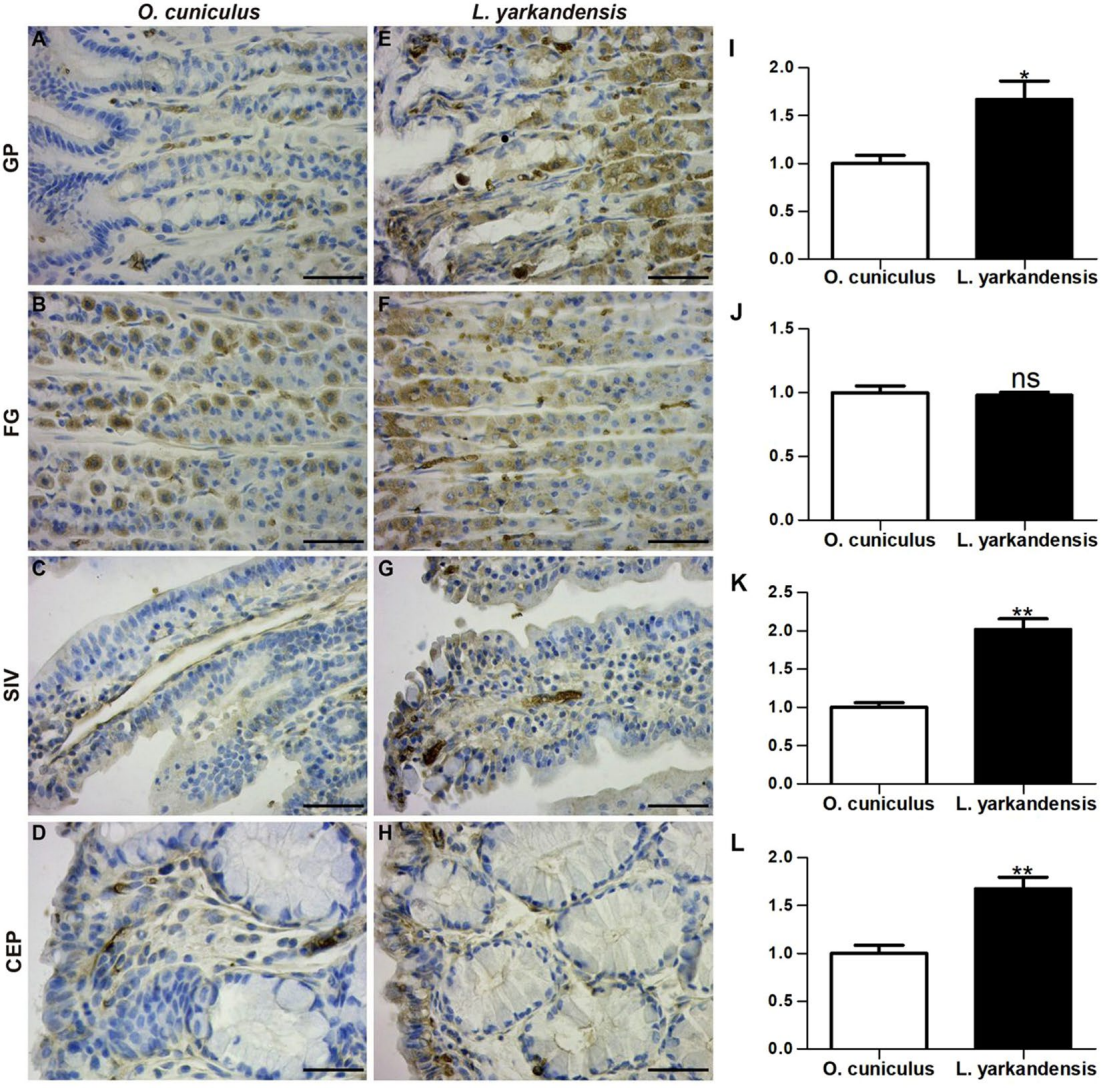
AQP1 distribution in the gastric pit (GP), fundic gland (FG), small intestinal villus (SIV), and colonic epithelium (CEP) in tissue sections from *O. cuniculus* (**A–D**) and *L. yarkandensis* (**E–H**). (**A–H**) Representative immunohistochemistry of the AQP1 distribution in the gastric pit, fundic gland, small intestinal villus, and colonic epithelium of *O. cuniculus* and *L. yarkandensis*. Paraffin sections (6 μm) of gastrointestinal tract tissue from *O. cuniculus* and *L. yarkandensis*. Sections were incubated with an anti-AQP1 antibody. Scale bar for **A**–**H**: 50 μm. (**I–L**) Densitometric analysis of all immunohistochemical results for the gastric pit, fundic gland, small intestinal villus, and colonic epithelium from *O. cuniculus* and *L. yarkandensis* (n = 6 animals per group). Ns: *p* > 0.05; *p < 0.05; **p < 0.01. In *O. cuniculus*, weak labelling was detected in the gastric pit (**A**), small intestinal villus (**C**), and colonic epithelium (**D**). In contrast, strong labelling was detected in endothelial cells of capillaries in the surrounding gastric pit (**E**), central lacteal cells in the small intestinal villus (**G**), and surface-absorptive cells in the colonic epithelium (**H**) of *L. yarkandensis*. The densitometric values show higher expression levels of AQP1 in the gastric pit (**I**), small intestinal villus (**K**), and colonic epithelium (**L**) of *L. yarkandensis* than in *O. cuniculus* and a lower level of AQP1 in the fundic gland (**J**) of *L. yarkandensis* than in *O. cuniculus*.

King and colleagues^4^ demonstrated that AQPs are a family of highly conserved water-specific membrane-channel proteins. AQP1, AQP3 and AQP4 mRNA and amino acid sequences were available in GenBank (Table 1). Furthermore, amino acid sequence alignment showed that the AQP1, AQP3 and AQP4 amino acid sequences were 99%, 98% and 99% identical, respectively, between *O. cuniculus* and *L. yarkandensis*. Immunohistochemistry was performed to analyse the localization of AQP1, AQP3 and AQP4 in the gastrointestinal tract of *O. cuniculus* and *L. yarkandensis*. AQP1 staining was localized in endothelial cells of capillaries in the surrounding gastric pit (Fig. 4A,E), parietal cells of the fundic gland (Fig. 4B,F), central lacteal cells of the small intestinal villus (Fig. 4C,G), and surface-absorptive cells of the colonic epithelium (Fig. 4D,H). Densitometric analysis of the immunohistochemical results revealed higher expression levels of AQP1 in the gastric pit, small intestinal villus, and colonic epithelium of *L. yarkandensis*—166 ± 19%, and 202 ± 14%, 168 ± 12% of those in *O. cuniculus*, respectively (n = 6 animals per group) (Fig. 4I,K,L)—and a decreased level of AQP1 in the fundic gland (Fig. 4J) of *L. yarkandensis*, 97 ± 2% of that in *O. cuniculus* (n = 6 animals per group).

**Table 1:** Sequences of primers for real-time PCR and GenBank accession numbers.

Gene	Sequence (5′–3′)	Accession number	
		*O. cuniculus*	*L. yarkandensis*
*β-actin*	F: TTTTGAATGGTCAGCCATCGT	NM_001101683	
	R: GAGACCAAAAGCCTTCATACATCT		
*AQP1*	F: GACTACACTGGCTGTGGCATTAAC	XM_017344416	MK947034
	R: GATCCAGTGGTTGTTGAAGTTGTG		
*AQP3*	F: GGATCAAGCTGCCCATCTACA	XM_002708029	MK947036
	R: CATCATAATATAGCCCGAAAACGA		
*ENaC*	F: CAGCGTTTTGCCTTGTTCAC	XM_002713474	MK947038
	R: GACACGTTCATGGGCAAGACT		
*Na*^+^*-K*^+^*-ATPase*	F: AATACGGAACGGACTTGAGC	NM_001082728	MN045869
	R: CCGACAGAACTTGACCCATT		

AQP, aquaporin; ENaC, epithelial sodium channel.

**Figure 5:**
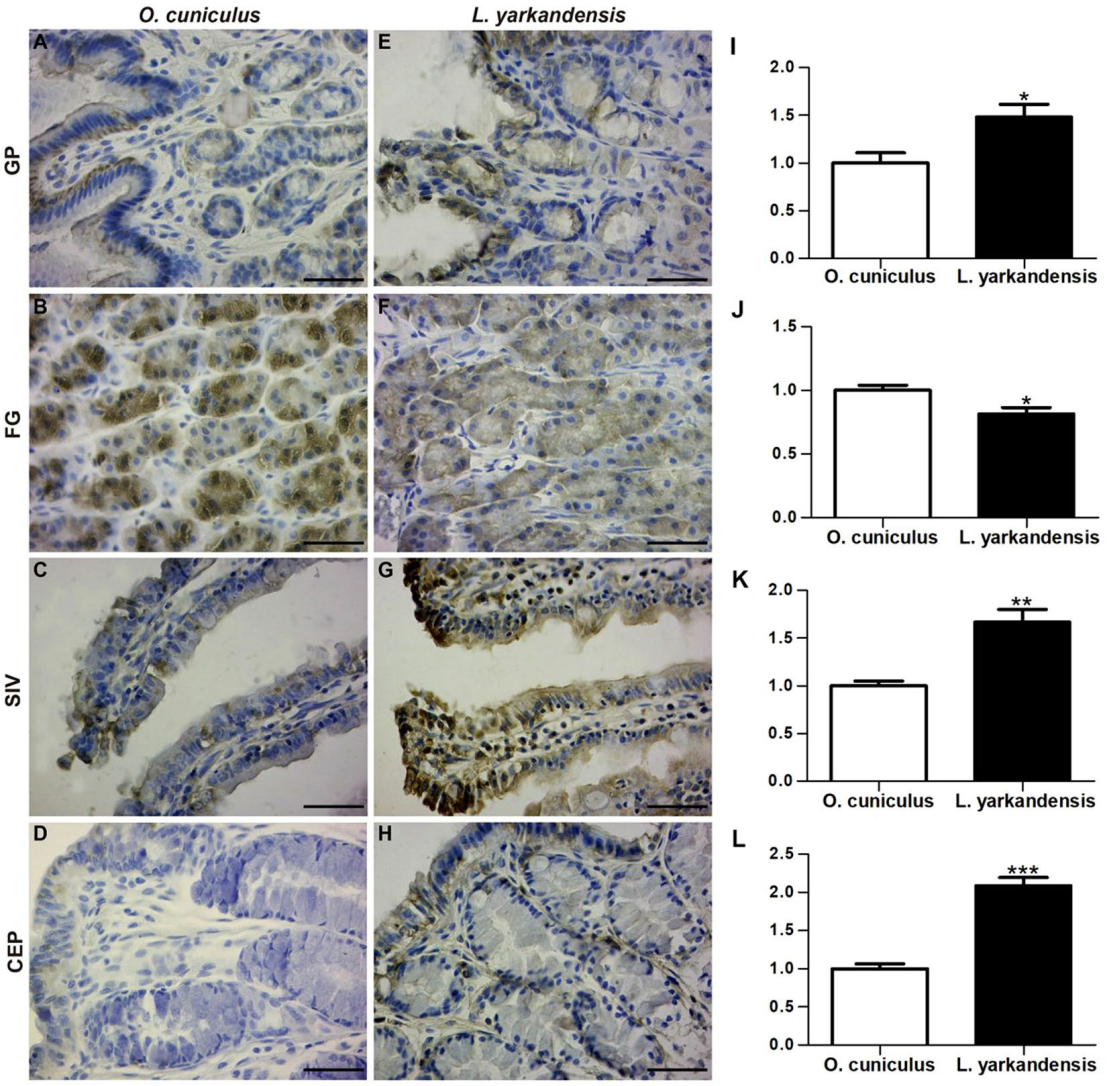
AQP3 distribution in the gastric pit (GP), fundic gland (FG), small intestinal villus (SIV), and colonic epithelium (CEP) in tissue sections from *O. cuniculus* (**A–D**) and *L. yarkandensis* (**E–H**). (**A–H**) Representative immunohistochemistry of the AQP3 distribution in the gastric pit, fundic gland, small intestinal villus, and colonic epithelium of *O. cuniculus* and *L. yarkandensis*. Paraffin sections (6 μm) of gastrointestinal tract tissue from *O. cuniculus* and *L. yarkandensis*. Sections were incubated with an anti-AQP3 antibody. Scale bar for A-H: 50 μm. (**I–L**) Densitometric analysis of all immunohistochemical results for the gastric pit, fundic gland, small intestinal villus, and colonic epithelium from *O. cuniculus* and *L. yarkandensis* (n = 6 animals per group). *p < 0.05; **p < 0.01; ***p < 0.001. In *O. cuniculus*, weak labelling was detected in the gastric pit (**A**), small intestinal villus epithelium (**C**), and colonic epithelium (**D**), and strong labelling was detected in parietal cells of the fundic gland (**B**). In contrast, strong labelling was detected in surface mucous cells of the gastric pit (**E**), surface-absorptive cells of the small intestinal villus (**G**) and the colonic epithelium (**H**) of *L. yarkandensis*, and weak labelling was detected in parietal cells of the fundic gland (**F**). The densitometric values showed higher expression levels of AQP3 in the gastric pit (**I**), small intestinal villus (**K**), and colonic epithelium (**L**) of *L. yarkandensis* than in *O. cuniculus*, and a lower level of AQP3 in parietal cells of the fundic gland (**J**) of *L. yarkandensis* than in *O. cuniculus*.

AQP3 staining was localized in surface mucous cells of gastric pit (Fig. 5A,E), parietal cells of the fundic gland (Fig. 5B,F), surface-absorptive cells of the small intestinal villus (Fig. 5C,G) and the colonic epithelium (Fig. 5D,H). Densitometric analysis of the immunohistochemical results revealed higher expression levels of AQP3 in the gastric pit, small intestinal villus, and colonic epithelium of *L. yarkandensis*—148 ± 13%, 166 ± 14%, and 209 ± 11% of those in *O. cuniculus*, respectively (n = 6 animals per group) (Fig. 5I,K,L)— and a lower level of AQP3 in the fundic gland of *L. yarkandensis*, 81 ± 5% of that in *O. cuniculus* (n = 6 animals per group) (Fig. 5J).

**Figure 6:**
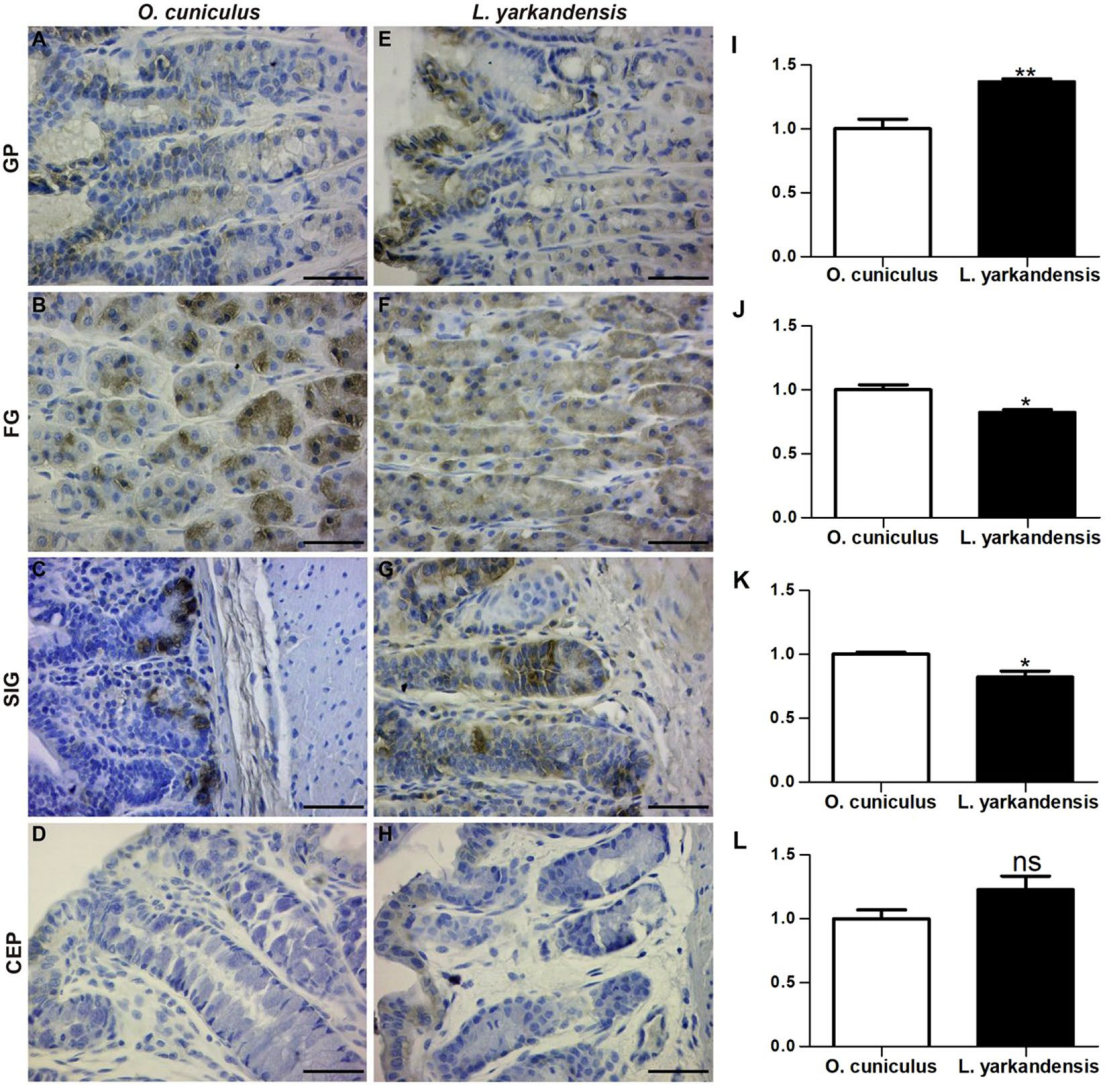
AQP4 distribution in the gastric pit (GP), fundic gland (FG), small intestinal gland (SIG), and colonic epithelium (CEP) in tissue sections from *O. cuniculus* (**A–D**) and *L. yarkandensis* (**E–H**). (**A–H**) Representative immunohistochemistry of the AQP4 distribution in the gastric pit, fundic gland, small intestinal gland, and colonic epithelium of *O. cuniculus* and *L. yarkandensis*. Paraffin sections (6 μm) of gastrointestinal tract tissue from *O. cuniculus* and *L. yarkandensis*. Sections were incubated with an anti-AQP4 antibody. Scale bar for A-H: 50 μm. (**I–L**) Densitometric analysis of all immunohistochemical results for the gastric pit, fundic gland, small intestinal gland, and colonic epithelium from *O. cuniculus* and *L. yarkandensis* (n = 6 animals per group). Ns: *p* > 0.05; *p < 0.05; **p < 0.01. In *O. cuniculus*, weak labelling was detected in the gastric pit **(A**), and strong labelling was detected in the parietal cells of the fundic gland (**B**) and small intestinal gland (**C**). In contrast, strong labelling was detected in surface mucous cells of the gastric pit (**E**) of *L. yarkandensis*, and weak labelling was detected in parietal cells of the fundic gland (**F**) and small intestinal gland (**G**). The densitometric values show higher expression levels of AQP4 in the gastric pit (**I**) of *L. yarkandensis* than in *O. cuniculus* and lower levels of AQP4 in parietal cells of the fundic gland (**J**) and small intestinal gland (**K)** of *L. yarkandensis* than in *O. cuniculus*.

AQP4 staining was localized in surface mucous cells of the gastric pit (Fig. 6A,E), parietal cells of the fundic gland (Fig. 6B,F), small intestinal gland cells (Fig. 6C,G) and the colonic epithelium (Fig. 6D,H). Densitometric analysis of the immunohistochemical results revealed higher expression levels of AQP4 in the gastric pit and colonic epithelium of *L. yarkandensis*—137 ± 2% and 123 ± 11% of those in *O. cuniculus*, respectively (n = 6 animals per group) (Fig. 6I,L)—and lower levels of AQP4 in the fundic gland and small intestinal gland of *L. yarkandensis*—82 ± 2% and 82 ± 5% of those in *O. cuniculus*, respectively (n = 6 animals per group) (Fig. 6J,K). Together, these results suggested that the expression levels of AQP1, AQP3 and AQP4 in the gastric pit, small intestinal villus and colonic epithelium, especially AQP1 and AQP3 in the colonic epithelium, are higher in *L. yarkandensis* than in *O. cuniculus*.

### Localization of the epithelial sodium channel and Na^+^-K^+^-ATPase in the *O. cuniculus* and *L. yarkandensis* gastrointestinal tracts

**Figure 7:**
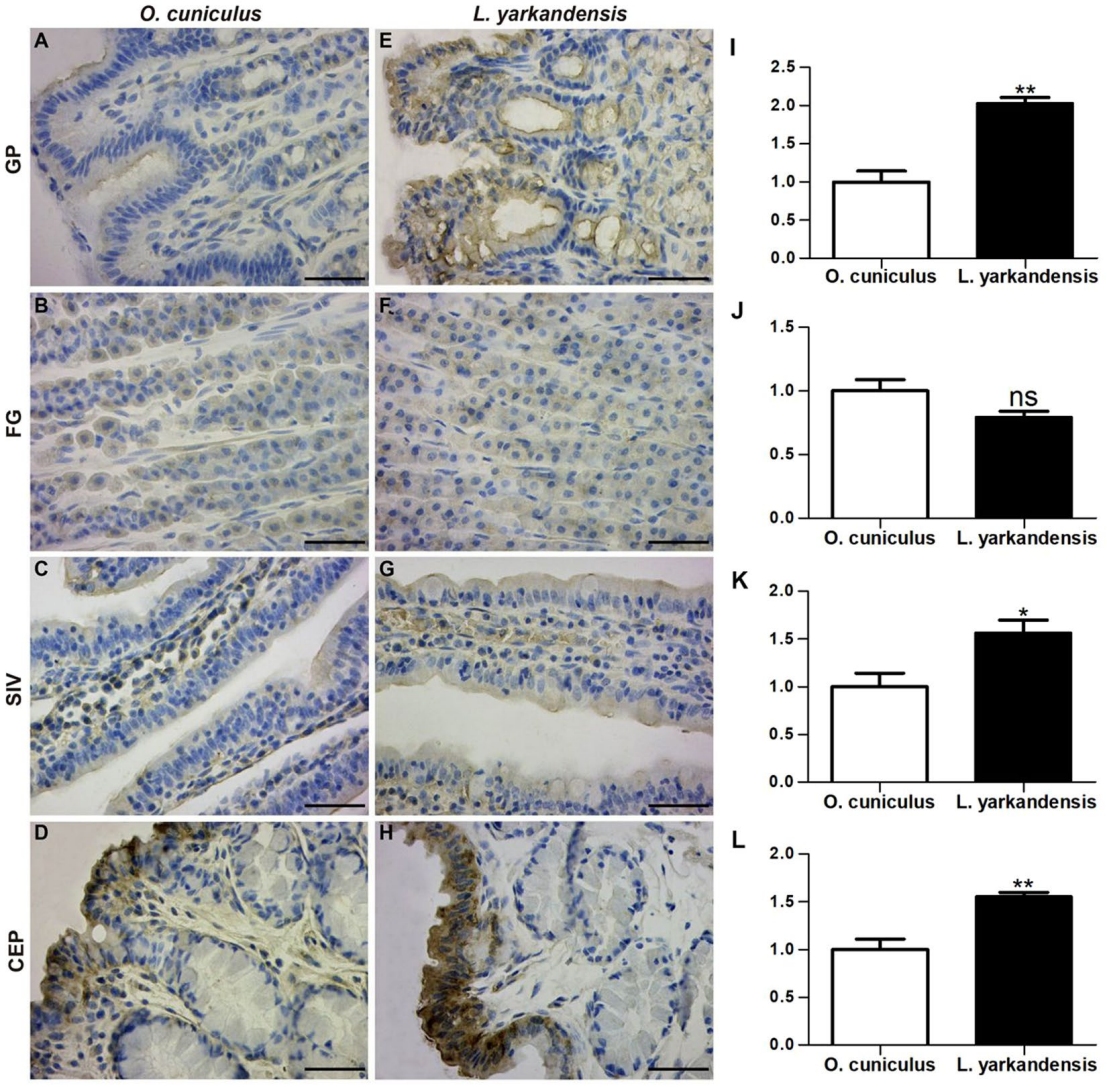
Epithelial sodium channel (ENaC) distribution in the gastric pit (GP), fundic gland (FG), small intestinal villus (SIV), and colonic epithelium (CEP) in tissue sections from *O. cuniculus* (**A–D**) and *L. yarkandensis* (**E–H**). (**A–H**) Representative immunohistochemistry of the ENaC distribution in the gastric pit, fundic gland, small intestinal villus, and colonic epithelium of *O. cuniculus* and *L. yarkandensis*. Paraffin sections (6 μm) of gastrointestinal tract tissue from *O. cuniculus* and *L. yarkandensis*. Sections were incubated with an anti-ENaC antibody. Scale bar for A-H: 50 μm. (**I–L**) Densitometric analysis of all immunohistochemical results for the gastric pit, fundic gland, small intestinal villus, and colonic epithelium from *O. cuniculus* and *L. yarkandensis* (n = 6 animals per group). Ns: *p* > 0.05; *p < 0.05; **p < 0.01. In *O. cuniculus*, weak labelling was detected in the gastric pit (**A**), small intestinal villus (**C**), and colonic epithelium (**D**), and strong labelling was detected in parietal cells of the fundic gland (**B**). In contrast, strong labelling was detected in surface mucous cells in the gastric pit (**E**), and surface-absorptive cells in the small intestinal villus (**G**) and colonic epithelium (**H**) of *L. yarkandensis*, and weak labelling was detected in parietal cells of the fundic gland (**F**). The densitometric values show higher expression levels of ENaC in the gastric pit (**I**), small intestinal villus (**K**), and colonic epithelium (**L**) of *L. yarkandensis* than in *O. cuniculus* and lower levels of ENaC in parietal cells of the fundic gland (**J**) of *L. yarkandensis* than in *O. cuniculus*.

Water absorption through AQPs is driven by an osmotic gradient that is generated by transcellular Na^+^ transport. Na^+^ entry is conductive and mediated by the apically located epithelial sodium channel (ENaC), and Na^+^ exit is mediated through the basolateral Na^+^-K^+^-ATPase. Therefore, we investigated the localization of ENaC and Na^+^-K^+^-ATPase in the *O. cuniculus* and *L. yarkandensis* gastrointestinal tracts. ENaC and Na^+^-K^+^-ATPase mRNA and amino acid sequences were available in GenBank (Table 1). Amino acid sequence alignment showed 98% sequence identity in the ENaC and Na^+^-K^+^-ATPase amino acid sequences between *O. cuniculus* and *L. yarkandensis*. ENaC staining was localized in surface mucous cells of the gastric pit (Fig. 7A,E), parietal cells of the fundic gland (Fig. 7B,F), surface-absorptive cells of the small intestinal villus epithelium (Fig. 7C,G) and the colonic epithelium (Fig. 7D,H). Densitometric analysis of the immunohistochemical results revealed higher expression levels of ENaC in the gastric pit, small intestinal villus, and colonic epithelium of *L. yarkandensis*—203 ± 8%, 156 ± 14%, 155 ± 5% of those in *O. cuniculus*, respectively (n = 6 animals per group) (Fig. 7I,K,L)—and lower levels of ENaC in the fundic gland of *L. yarkandensis*, 79 ± 5% of that in *O. cuniculus* (n = 6 animals per group) (Fig. 7J).

**Figure 8:**
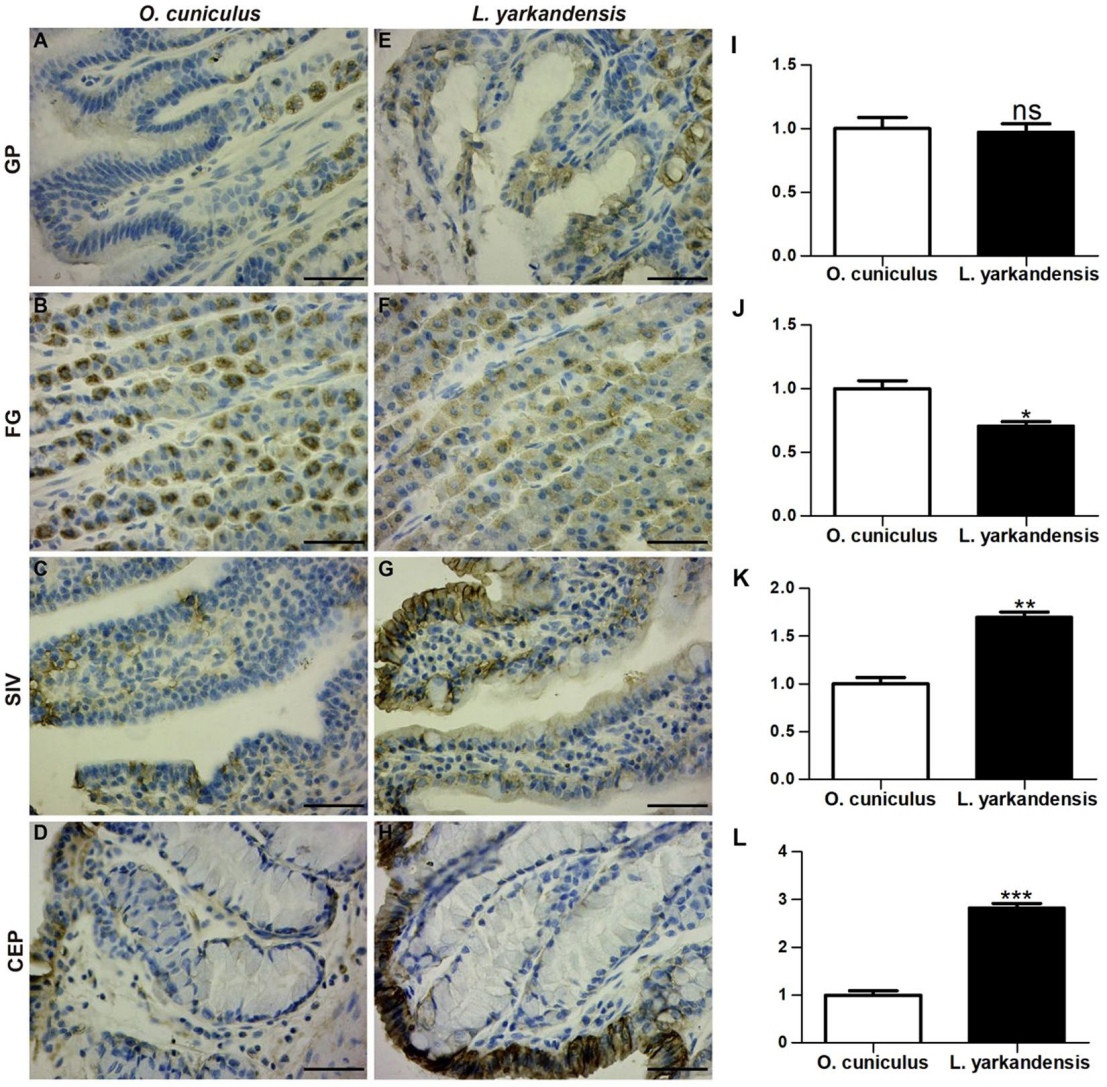
Na^+^-K^+^-ATPase distribution in the gastric pit (GP), fundic gland (FG), small intestinal villus (SIV), and colonic epithelium (CEP) in tissue sections from *O. cuniculus* (**A–D**) and *L. yarkandensis* (**E–H**). (**A–H**) Representative immunohistochemistry of the Na^+^-K^+^-ATPase distribution in the gastric pit, fundic gland, small intestinal villus, and colonic epithelium of *O. cuniculus* and *L. yarkandensis*. Paraffin sections (6 μm) of gastrointestinal tract tissue from *O. cuniculus* and *L. yarkandensis*. Sections were incubated with an anti-Na^+^-K^+^-ATPase antibody. Scale bar for A-H: 50 μm. (**I-L**) Densitometric analysis of all immunohistochemical results for the gastric pit, fundic gland, small intestinal villus, and colonic epithelium from *O. cuniculus* and *L. yarkandensis* (n = 6 animals per group). Ns: *p* > 0.05; *p < 0.05; **p < 0.01; ***p < 0.001. In *O. cuniculus*, basolateral labelling was very weak/absent in the gastric pit (**A**), strong labelling was detected in parietal cells of the fundic gland (**B**), and weak labelling was detected in the small intestinal villus (**C**) and colonic epithelium (**D**). In contrast, weak labelling was detected in parietal cells of the fundic gland (**F**), and strong labelling was detected in the basolateral membranes of surface-absorptive cells in the small intestinal villus (**G**) and colonic epithelium (**H**) of *L. yarkandensis*. The densitometric values show no difference in the Na^+^-K^+^-ATPase protein abundance in the gastric pit (**I**) between *O. cuniculus* and *L. yarkandensis* but lower Na^+^-K^+^-ATPase protein abundance in parietal cells of the fundic gland (**J**) and higher Na^+^-K^+^-ATPase protein abundance in the small intestinal villus (**K**) and colonic epithelium (**L**) of *L. yarkandensis* than those in *O. cuniculus*.

Na^+^-K^+^-ATPase staining was localized in surface mucous cells of the gastric pit (Fig. 8A,E), parietal cells of the fundic gland (Fig. 8B,F), and surface-absorptive cells in the small intestinal villus epithelium (Fig. 8C,G) and colonic epithelium (Fig. 8D,H). Densitometric analysis of the immunohistochemical results revealed higher expression levels of Na^+^-K^+^-ATPase in the small intestinal villus and colonic epithelium of *L. yarkandensis*—170 ± 6% and 282 ± 10% of those in *O. cuniculus*, respectively (n = 6 animals per group) (Fig. 8K,L)—and lower levels of Na^+^-K^+^-ATPase in the fundic gland of *L. yarkandensis*, 71 ± 3% of that in *O. cuniculus* (n = 6 animals per group) (Fig. 8J). Together, these results indicated that the levels of epithelial sodium channel and Na^+^-K^+^-ATPase expression in the small intestinal villus and colon epithelium were higher in *L. yarkandensis* than in *O. cuniculus*.

### mRNA expression levels of AQP1, AQP3, epithelial sodium channel and Na^+^-K^+^-ATPase in the *O*. *cuniculus* and *L. yarkandensis* colon

**Figure 9:**
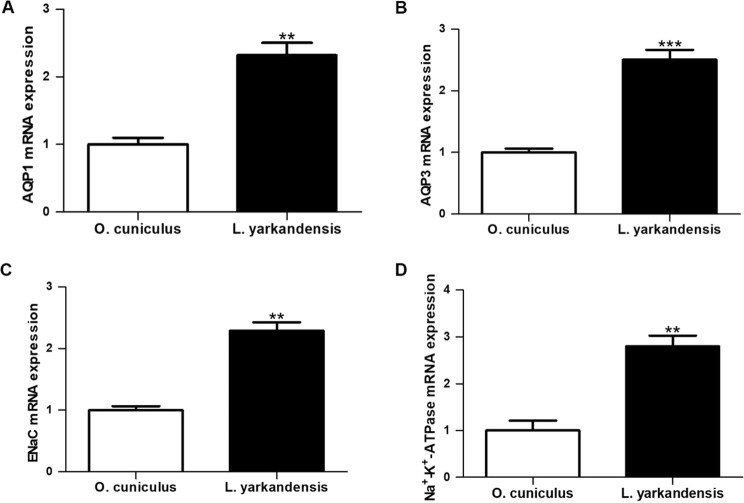
*AQP1*, *AQP3, ENaC and Na*^+^*-K*^+^*-ATPase* mRNA expression in the colon of *O. cuniculus* and *L. yarkandensis*. Total RNAs were extracted from the renal medulla (*n* = 6 animals per group), and 1.0 μg/samples were subjected to the RT reaction followed by PCR with primers specific for *AQP1, AQP3, ENaC and Na*^+^*-K*^+^*-ATPase*, and *β-actin*, respectively. The values were quantified as a ratio of the expression of each gene normalized for the expression level of *β-actin* for each sample as an internal loading control. Values were presented as fraction of the mean *O. cuniculus* values. *P* values refer to the comparison of *L. yarkandensis* values with *O. cuniculus* values. (**A**) Quantitative RT-PCR analysis of *AQP1* mRNA in the colon of *O. cuniculus* and *L. yarkandensis*. (**B**) Quantitative RT-PCR analysis of *AQP3* mRNA in the colon of *O. cuniculus* and *L. yarkandensis*. (**C**) Quantitative RT-PCR analysis of *ENaC* mRNA in the colon of *O. cuniculus* and *L. yarkandensis*. (**D**) Quantitative RT-PCR analysis of *Na*^+^*-K*^+^*-ATPase* mRNA in the colon of *O. cuniculus* and *L. yarkandensis*. Quantitative RT-PCR showed that *AQP1, AQP3, ENaC and Na*^+^*-K*^+^*-ATPase* mRNA levels were higher in the colon of *L. yarkandensis* than in *O. cuniculus*. ***p <0.001, **p < 0.01.

Immunohistochemistry data concluded that AQP1, AQP3, ENaC and Na^+^-K^+^-ATPase proteins were higher expression levels in the colon of *L. yarkandensis* than those of *O. cuniculus*. It is necessary to investigate whether the higher expression of AQP1, AQP3, ENaC and Na^+^-K^+^-ATPase protein abundance was in parallel with their mRNA in the colon of *L. yarkandensis*. The nucleotide sequence alignment showed that the identity of AQP1 nucleotide sequence among *O. cuniculus* and *L. yarkandensis* was 99%, and primer-BLAST showed AQP1 primer that was specific to *O. cuniculus* and *L. yarkandensis* AQP1. The alignment results of AQP3, ENaC and Na^+^-K^+^-ATPase were similar to AQP1. So, we performed quantitative RT-PCR to determine the levels of *AQP1*, *AQP3*, *ENaC* and *Na*^+^*-K*^+^*-ATPase* mRNA expression in the colon of *O. cuniculus* and *L. yarkandensis*. Quantitative RT-PCR suggested that higher expression levels *of AQP1*, *AQP3*, *ENaC* and *Na*^+^*-K*^+^*-ATPase* mRNA in the colon of *L. yarkandensis* —232 ± 18%, 250 ± 16%, 229 ± 13% and 277 ± 20% of those in *O. cuniculus*, respectively (n = 6 animals per group) (Fig. 9A–D). Thus, these results were in concordance with immunohistochemistry results.

### Water Content of *O. cuniculus* and *L. yarkandensis* Faeces

**Figure 10:**
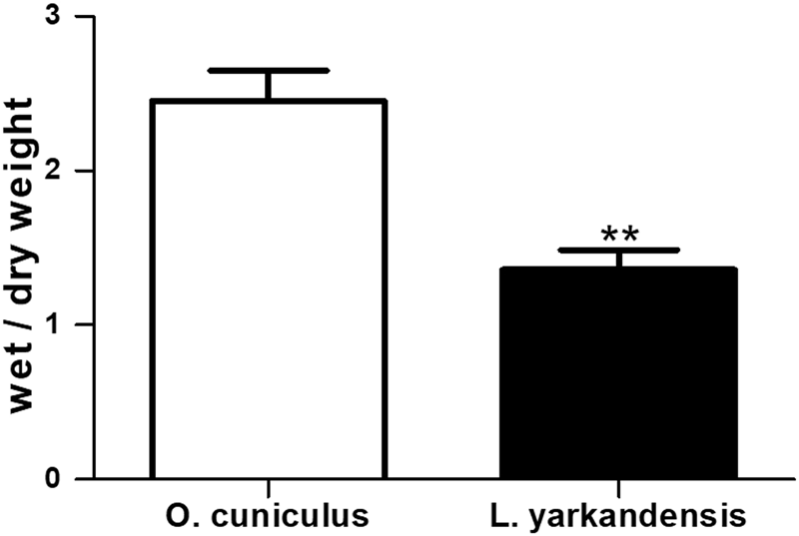
Lower water content in *L. yarkandensis* faeces. Wet/dry weight ratios in whole faeces from *O. cuniculus* and *L. yarkandensis* were determined. The values are expressed as grams of wet weight/grams of dry weight (n = 6 animals per group). **p < 0.01.

To quantitatively assess the water content of *O. cuniculus* and *L. yarkandensis* faeces, whole-faeces wet/dry weight ratios were determined. The ratio of wet/dry weights for whole faeces was lower for *L. yarkandensis* (136 ± 13%, n = 6 animals per group, *P* <0.01) than for *O. cuniculus* (246 ± 20%, n = 6 animals per group) (Fig. 10).

## Discussion

Haematoxylin and eosin staining showed that the components of the *L. yarkandensis* stomach include the mucosa, submucosa, muscle layer, and serosa and that there are obvious longitudinal folds and gastric pits. The mucosal epithelium is a single-layered columnar epithelium composed mainly of cells with a tall columnar shape and a nucleus located at the base. There are many gastric pits on the mucosal surface, enhancing the dilatability and digestive capacity of the stomach. Haematoxylin and eosin staining also showed that the duodenum of *L. yarkandensis* has developed intestinal villi, which are broad and leafy, and duodenal glands. These results suggested that the duodenum of *L. yarkandensis* is not only the part that absorbs nutrients but also the part that digests food. Histology of the large intestine of *L. yarkandensis* showed that its large intestinal gland is very developed, long and straight, with many goblet cells.

Next, we detected the distribution of AQPs in the stomach, small intestine and large intestine of *O. cuniculus* and *L. yarkandensis*. We found the locations of AQP1 in the colonic epithelium, central lacteal cells, fundic gland parietal cells, and capillary endothelial cells of *O. cuniculus* and *L. yarkandensis*. The distribution of AQP1 in the colonic epithelium indicated that it is involved in transepithelial water transport, as has been revealed for the proximal tubules and descending thin limb segments of the mammalian nephron^35,36^. A role for AQP1 as a water channel in fluid absorption in the colon corresponds to the significant decrease in the fluid absorption rate in the presence of *p*-chloromercuribenzenesulfonic acid (a mercurial agent)^37^. AQP1 was located in intestinal lacteals and capillaries, implying a role in the water permeability of lymphatics and capillary beds^38^. The present experiments do not clearly indicate that AQP1 is expressed on parietal cells; however, in this study, AQP1 was found in fundic gland parietal cells, which may imply a role in gastric acid secretion. Our results revealed that the expression levels of AQP1 in the colonic epithelium and central lacteal were higher in *L. yarkandensis* than in *O. cuniculus*, suggesting that the water permeability of the colonic epithelium, lymphatics and capillary beds is higher in *L. yarkandensis*.

We found the distribution of AQP3 in the colonic epithelium, small intestinal villus epithelium, gastric pits and fundic glands of *O. cuniculus and L. yarkandensis*. The locations of AQP3 in the colonic epithelium were consistent with those described in human and rat colons, where AQP3 was detected in the basolateral membranes of colonic epithelial cells^39–42^. The presence of AQP3 in epithelial cells may indicate its role in transepithelial water transport. Previous studies have shown that AQP3 plays an important role in water absorption in the colon^43–45^. The presence of AQP3 in the fundic gland may suggest its role in gastric fluid secretion^12,46^. Our results suggested that the expression levels of AQP3 in the colonic epithelium and small intestinal villus epithelium were higher in *L. yarkandensis* than in *O. cuniculus*. Ikarashi and colleagues showed a decrease in the AQP3 expression level in the colon, which inhibited water absorption from the luminal side to the vascular side^47,48^. Based on these experiments, we can conclude that the colon of *L. yarkandensis* has a higher ability for transepithelial water absorption.

We found the locations of AQP4 in the fundic gland, small intestinal gland and colonic epithelium of *O. cuniculus* and *L. yarkandensis*. To date, AQP4 has been detected in the stomach. Experiments with rats demonstrated that AQP4 was localized to the basolateral membrane of fundic gland parietal cells^49^. Subsequently, experiments with humans suggested that AQP4 was cloned from the stomach and localized to both parietal cells and chief cells of the fundic gland^50^. It has been postulated that AQP4 participates in gastric fluid secretion. However, experiments with AQP4 knockout mice revealed no effect of AQP4 deletion on gastric fluid secretion, and the results provided direct evidence against a role of AQP4 in gastric fluid secretion^51^. Our results showed that weak labelling was detected in the colonic epithelium of *O. cuniculus* and *L. yarkandensis*, in agreement with previous findings in transgenic null mice, in which there was little or no effect of AQP4 deletion on colonic fluid transport or faecal dehydration^30^.

Water absorption through AQPs is driven by an osmotic gradient that is generated by transcellular Na^+^ transport. Apical Na^+^ entry in surface-absorptive cells of the colonic epithelium is mediated by the ENaC, and basolateral Na^+^ exit is mediated through the Na^+^-K^+^-ATPase, sodium transporters that were identified in the mammalian colon and lung at the mRNA and protein levels^31,52^. We found the ENaC and Na^+^-K^+^-ATPase distribution in the colonic epithelium, small intestinal villus epithelium, gastric pit and fundic gland, implying the roles of these transporters in Na^+^ absorption in the colon and small intestinal villus epithelium^31^. This Na^+^ absorption may provide the osmotic gradient for water absorption across both membranes of epithelial cells through apical AQP1 and basolateral AQP3 and AQP4.

We found a lower water content in *L. yarkandensis* faeces than in *O. cuniculus* faeces, suggesting that *L. yarkandensis* had a high capacity for faecal dehydration. This finding is consistent with the observation that animals that inhabit the desert exhibit physiological and morphological adaptations to arid environments, for example, high-concentration urine production and faecal dehydration^53^. Experiments with *Octodon degus*, a desert rodent, demonstrated that the colon of *O. degus* had a higher capacity for faecal dehydration than the rat colon^37^. Experiments with rats administered HgCl_2_ (an AQP3 functional inhibitor) demonstrated that the faecal water content in the HgCl_2_ administration group markedly increased to approximately 4-fold that in the control group^54^. Our results revealed that the expression levels of AQP3 in the colon were higher in *L. yarkandensis* than in *O. cuniculus* indicating that the colon of *L. yarkandensis* has a higher capacity for faecal dehydration.

We found that the expression levels of AQPs in the stomach, small intestine and colon were different. The low AQP expression in the stomach and small intestine was consistent with functional data in vesicles derived from these tissues, suggesting low plasma membrane water permeability^40^. The colonic epithelium is a tight epithelium with substantially higher electrical resistance and probably a much lower paracellular water permeability than the small intestinal epithelium^7^. *L. yarkandensis* had higher expression levels of AQP1 and AQP3 in colonic epithelium than *O. cuniculus*, which could contribute to the extraction of water from faeces to produce dehydrated faecal matter. The higher expression levels of AQP1 and AQP3 in colonic epithelium was more likely due to *L. yarkandensis* living in an arid desert environment for a long time. Ambient pressure can accelerate the rate of evolution of specific stress-sensitive proteins, produce new functions for specific environments or enhance existing functions, and improve animal fitness for this stressful environment^55^. For example, many studies showed that chronic cold exposure caused endotherms increased intestinal nutrients intake to meet increased energy demand for maintaining thermal homeostasis^56–58^. And the expression of digestive features that approximately match digestive capacities with dietary loads^59,60^. Furthermore, we also found higher levels of AQP1 and AQP3 in the kidneys of *L. yarkandensis*^61^. This may be related to the strategy of the *L. yarkandensis* to conserve body water.

**Figure 11:**
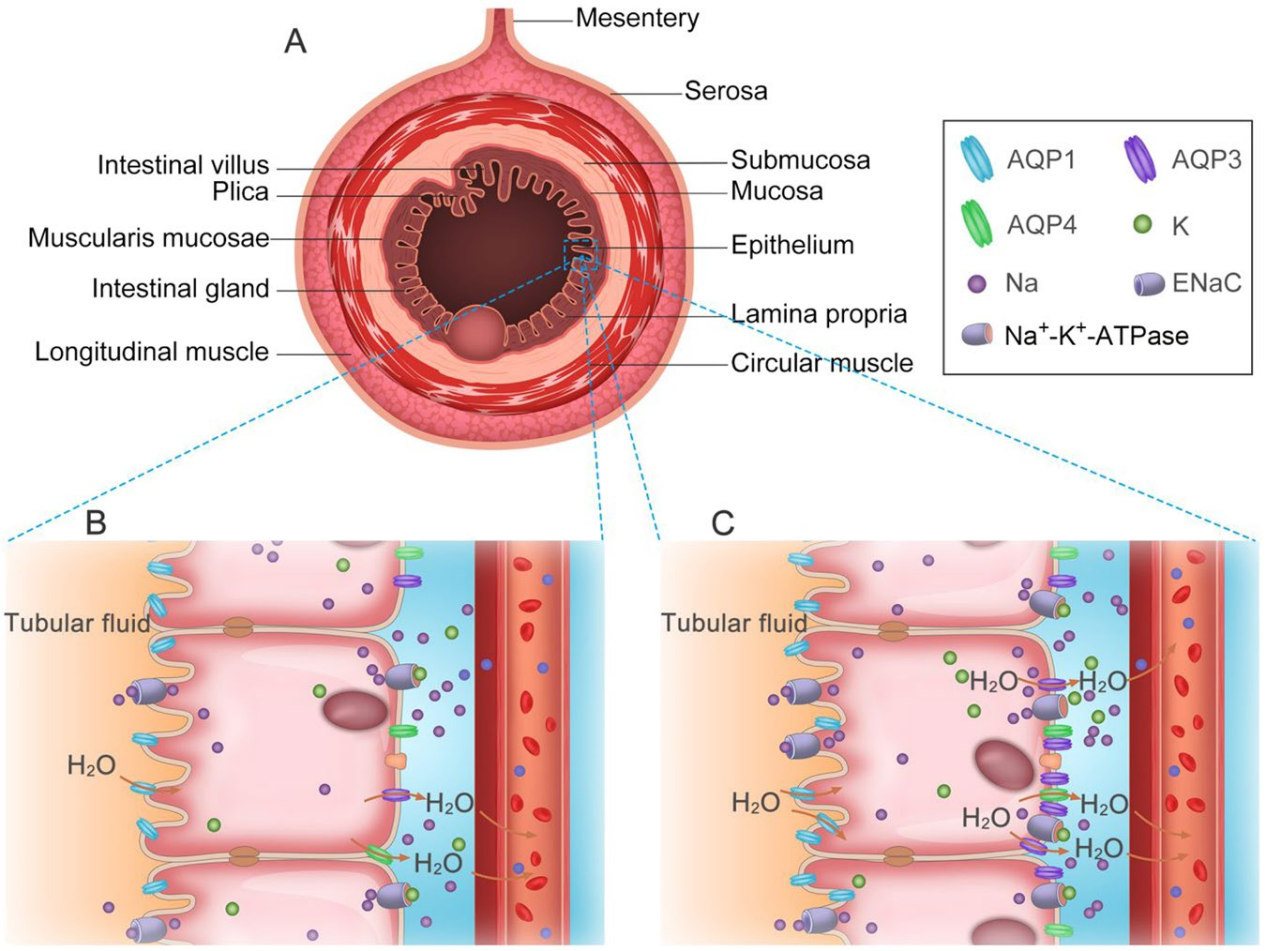
Schematic diagram illustrating the distribution of AQP1, AQP3, AQP4 and sodium transporters in the digestive tract of *O. cuniculus* and *L. yarkandensis*. (**A**) Structural diagram of the digestive tract. (**B**) The localization of AQP1, AQP3, AQP4 and sodium transporters in the intestinal epithelium of *O. cuniculus*. (**C**) The localization of AQP1, AQP3, AQP4 and sodium transporters in the intestinal epithelium of *L. yarkandensis*. AQP1 (blue) is located in the apical membrane of intestinal epithelial cells. Both AQP3 (purple) and AQP4 (green) are present in the basolateral membrane of intestinal epithelial cells. ENaC is located in the apical membrane of intestinal epithelial cells. Na^+^-K^+^-ATPase is located in the basolateral membrane of the same cells.

In conclusion, the locations of AQP1, AQP3, AQP4 and sodium transporters in the gastrointestinal tract of *O. cuniculus* and *L. yarkandensis* to sum up in Fig. 11. In the gastrointestinal tract of *L. yarkandensis*, AQP1 was located in the colonic epithelium, central lacteal cells, fundic gland parietal cells, and capillary endothelial cells; AQP3 was located in the colonic epithelium, small intestinal villus epithelium, gastric pit and fundic gland; AQP4 was located in the fundic gland, small intestinal gland and colonic epithelium; and ENaC and Na^+^-K^+^-ATPase were located in the colonic epithelium, small intestinal villus epithelium, gastric pit and fundic gland. The dramatically higher expression levels of AQP3 in the colon of *L. yarkandensis* than in the colon of *O. cuniculus* revealed that the colon of *L. yarkandensis* had higher water permeability, and the higher levels of ENaC and Na^+^-K^+^-ATPase expression in the colon of *L. yarkandensis* provided an osmotic gradient for water absorption through AQPs. From these data, it can be concluded that the colon of *L. yarkandensis* has a higher capacity for faecal dehydration.

## Materials and Methods

### Animals and tissues

This study was carried out in male adult *O. cuniculus* and *L. yarkandensis* (1.5–1.8 kg). All experiments were performed according to international regulations for animal care and were approved by the Animal Care and Use Committee of Xinjiang Uygur Autonomous Region of China. Adult *O. cuniculus* was provided by the animal laboratory station of Tarim University. *L. yarkandensis* was collected from Shaya County, Aksu Prefecture, northwest of the Tarim Basin. And animals were assessed to be adult based on a skull length of greater than 75.50 mm. These animals were maintained initially in individual cages and had free access to food and drinking water at all times. One week after feeding, we collected the faeces of these animals to measure their water content; the animals were anaesthetized with 3% pentobarbital sodium (0.9 ml/kg). The stomach, duodenum, jejunum, ileum, caecum, colon and rectum (the length of each segment was approximately 0.5 cm) were removed and placed in ice-cold 0.85% sodium chloride solution to remove the contents and were then inflated with 4% paraformaldehyde (Sigma-Aldrich, Shanghai, China) and fixed overnight for HE staining and immunohistochemical examination^61^. And some tissues were stored in RNA preservation solutions for analysis of RNA levels.

### Wet/dry weight ratios of faeces

Faecal samples were collected from *L. yarkandensis* and *O. cuniculus* (n = 6 animals per group) and weighed to obtain the “wet” faecal weights. These faeces were then placed in a 60 °C oven with desiccant and weighed after 4 to 6 d. The “dry” faecal weights were recorded after the weights no longer changed on successive days. The ratio of the wet weight to the dry weight of the faeces was calculated as the wet weight obtained by weighing divided by the dry weight.

### Haematoxylin and eosin staining

We used haematoxylin and eosin staining to observe the histological structure of the stomach, small intestine and large intestine. Fixed gastrointestinal tissues were dehydrated in a gradient alcohol series, cleared with xylene, and embedded in paraffin. Paraffin-embedded blocks were fixed on a Lycra paraffin slicer (Leica RM2125RTS, Shanghai, China) for serial sectioning (6 μm thick). Sliced slides were deparaffinized with xylene and hydrated with a gradient alcohol series. Sections were stained with haematoxylin (Sigma-Aldrich, Shanghai, China) for 1 min to 2 min, rinsed with running water for 20 min, stained with 0.5% eosin (Sigma-Aldrich, Shanghai, China) for 30 s, dehydrated in a gradient alcohol series, cleared with xylene, and used for histological observation and imaging (Motic BA600, Beijing, China) after sealing.

### Immunohistochemistry

Localization of the AQP1, AQP3, AQP4, epithelial sodium channel (ENaC) and Na^+^-K^+^-ATPase proteins was evaluated in fixed gastrointestinal tissues of *L. yarkandensis* and *O. cuniculus* by immunocytochemistry. Immunocytochemical studies were performed in Paraffin-embedded gastrointestinal tissue, previously fixed in 4% paraformaldehyde. The experimental steps of immunohistochemistry have been described elsewhere^61^. The primary antibodies used were as follows: anti-AQP1, anti-AQP3, and anti-AQP4 (diluted to 4.0 μg/ml; Proteintech) and anti-ENaC and anti-Na^+^-K^+^-ATPase (diluted to 3.0 μg/ml; Proteintech). The acquired images were analysed in IpWin32 software^61^.

### Quantitative real-time PCR

Reverse-transcribed cDNA products were amplified by polymerase chain reaction (PCR) with primers specific for *AQP1, AQP3, AQP4, ENaC, Na*^+^*-K*^+^*-ATPase* and *β-actin* (Table 1). The steps for quantitative RT-PCR were described elsewhere^61^.

### Statistical analyses

GraphPad Prism statistical analysis software analysed of wet/dry weights, AQP1/*β*-actin, AQP3/*β*-actin, AQP4/*β*-actin, ENaC/*β*-actin and Na^+^-K^+^-ATPase/*β*-actin density ratios for RNA expression. Results are expressed as mean (M) ± standard error (SE). A *P*-value of <0.05 was considered statistically significant.
